# Editorial: Metabolic factors in erectile dysfunction

**DOI:** 10.3389/fendo.2023.1344191

**Published:** 2023-12-20

**Authors:** Xianghu Meng, Ke Rao, Jun Chen

**Affiliations:** ^1^ Department of Urology, The First Affiliated Hospital of Nanjing Medical University, Nanjing, Jiangsu, China; ^2^ Department of Urology, Tongji Hospital, Tongji Medical College, Huazhong University of Science and Technology, Wuhan, Hubei, China; ^3^ Department of Infertility and Sexual Medicine, the Third Affiliated Hospital of Sun Yat-sen University, Guangzhou, Guangdong, China

**Keywords:** erectile dysfunction, diabetes mellitus, hypertension, obesity, metabolic syndrome, chronic liver disease

Erectile dysfunction (ED) is a common male sexual dysfunction that seriously affects the life quality of patients. The worldwide prevalence of ED is predicted to reach 322 million cases by 2025 (1). The causes of ED are mainly divided into vasculogenic, neurogenic, endocrinological, drug-induced depression, systemic diseases and general ill health, local penile factors, or psychological problem. Among these causes, the increasing incidence of obesity, diabetes mellitus (DM), hyperlipidemia, hypertension, cardiovascular diseases, and metabolic syndrome has prompted studies to focus on the relationship between metabolic factors and erectile function.

Metabolic factors such as metabolic syndrome, cardiovascular disease, and obesity are risk factors for ED (2). Meanwhile, ED is a marker for metabolic conditions, preceding adverse metabolic events by several years. Therefore, healthcare providers encountering ED should screen for cardiovascular diseases or metabolic syndrome. Causality and associated mechanisms are difficult to establish because ED and these metabolic conditions also share risk factors such as age, hypertension, DM, insulin resistance, smoking, and increased BMI. Hence, the journal has organized this Research Topic: *Metabolic Factors and Erectile Dysfunction*.

In the Research Topic, a total of 13 articles were published, including 8 original research articles and 5 reviews. It involves metabolic factors such as DM, age, hypothalamic–pituitary–adrenal axis activity, serum 25(OH)D levels, idiopathic pulmonary fibrosis and chronic liver disease, and their mechanisms associated with erectile function.

As one of the important causes of ED, DM has attracted the attention of researchers. There are two studies focusing on DM-ED of the 8 original articles. A cross-sectional study from Ethiopia revealed that up to 83.8% of patients with ED have different levels of ED. Thus, they suggested that routine screening and management for ED in patients with DM should be part of the routine medical care, particularly for adult male patients and those with poor glycemic control. Another cross-sectional study comparing the characteristics between DM-induced erectile dysfunction (DM-ED) and non-DM-induced erectile dysfunction Chinese populations revealed that age, height, BMI, fasting blood glucose, follicle-stimulating hormone, triglycerides, total testosterone, and triglyceride-glucose index significantly differed between the DMED and non-DMED populations. Therefore, the above-mentioned metabolic factors play an important role in the occurrence of DM-ED.

In the study on age-related erectile dysfunction (A-ED), Zhou et al. used transcriptome analyses and bioinformatics methods to predict the possible lncRNA-miRNA-mRNA regulatory network that regulated the occurrence of ED in the elderly. Then they concluded that LncRNA ENSRNOT00000029245 possibly regulated downstream mRNAs leading to apoptosis in the cavernous tissue, fibrosis, and endothelial dysfunction, which ultimately caused ED. These findings provide seminal insights into the molecular biology of A-ED, which could spur the development of effective therapeutics.

Psychogenic erectile dysfunction (pED) is also an important type of ED. Psychological stress and its two stress response systems, the hypothalamic-pituitary-adrenal (HPA) axis and the autonomic nervous system (ANS), are closely related to pED. Xu et al. first studied the changes in perceived stress and two neural stress systems in pED patients. Their results suggested that the interrelation between ANS and HPA axis activity might enhance our comprehension of how stress affected the physical and mental health of pED patients.

And in other studies of ED, Yuan et al. uncovered the genetic links of ED and chronic prostatitis/chronic pelvic pain syndrome (CP/CPPS). Finally, NQO1 was identified as a key genetic link between ED and CP/CPPS. It was predominately enriched in corpus cavernosum endothelial cell, and correlated with other male urogenital and immune system diseases tightly. They identified the genetic profiles as well as corresponding regulatory network underlying interaction between ED and CP/CPPS via multi-omics analysis. These findings expanded a new understanding for the molecular mechanism of ED with CP/CPPS.

Zhang et al. investigated the connection between serum 25(OH)D and carotid artery intima-media thickness (CIMT) in men with ED. The serum level of 25(OH)D and IIEF-5 score were positively correlated, while the CIMT values and IIEF-5 score were negatively correlated. And they concluded that the level of serum 25(OH)D should be analyzed in men with ED, especially in patients with vasculogenic ED, and supplementation is recommended for those who were with vitamin D deficiency.

In a study on cisplatin, Yin et al. found that cisplatin treatment could induce ED, by affecting the content of endothelial and smooth muscle and causing the senescence-associated secretory phenotype in cavernous nerve (CN). Their results indicate that cisplatin treatment should be considered as a risk factor for ED. Clinicians should pay more attention to the erectile function of cancer patients who receive cisplatin treatment.

Several studies have found that ED is associated with interstitial lung disease. However, the causal relationship between idiopathic pulmonary fibrosis (IPF) and ED risk remains unclear. Zhang et al. conducted a two-sample Mendelian randomization (MR) study aiming to reveal the causal effect of IPF on ED risk and concluded that IPF may increase the ED risk of the European population.

In the five review papers, the main focus is on the research progress of pathological mechanism and treatment of ED. Song et al. presented a comprehensive overview of the current molecular pathogenesis of CN injury-induced ED. Song et al. introduced the physiological basis of erectile function and the pathophysiological changes in ED and summarized the current knowledge on the expression, biological functions, and molecular mechanisms of miRNAs in ED, especially the potential of miRNA-targeted therapies to improve ED. Pang et al. reviewed the diagnosis of ED and related physical therapy methods, and explored the pathogenesis of ED. In their opinion, these treatment methods could help many ED patients recover fully or partially from ED within the next few decades. A review conducted by Wang et al. summarized the recent advances on exosome therapy with animal models of ED, and proposed the prospect of future research in order to provide a basis for clinical trials and clinical translation. These reviews can help us better understand the occurrence, development and treatment progress of ED.

In addition to the above reviews, there is a literature review by Zang et al. explored the relationship between chronic liver diseases (CLDs) and ED. As we all know, liver is an important metabolic organ in the human body and is involved in the regulation of many metabolic factors. CLDs frequently result in the abnormal metabolism of sex hormones, glucose, and lipids and mental and psychological illnesses, all of which are significant risk factors for ED. The prevalence of ED in male patients with CLDs ranges from 24.6% to 85.0%. The mechanism by which liver damage affects penile erectile function is not fully understood. The review concluded that male patients with CLDs often have decreased testosterone levels, increased estrogen levels, and other sex hormone metabolism disorders, which may be reasons for the decline of erectile function.

However, metabolic factors are not limited to the factors discussed in our Research Topic. Other metabolic factors still need to be explored, such as high lipid levels, high prolactin, and thyroid hormone levels. In addition, whether ED is caused by a single metabolic factor or a variety of metabolic factors must be further elaborated. Finally, even though researchers performed a deep exploration about the Research Topic, the exact mechanics between metabolic factors and ED remains to be further studied.

## Author contributions

XM: Writing – original draft, Writing – review & editing. KR: Writing – review & editing. JC: Writing – review & editing.
